# Emerging mechanisms of microplastic-induced skin diseases: a perspective from the gut–skin axis

**DOI:** 10.1186/s12967-025-07300-w

**Published:** 2026-02-16

**Authors:** Xueer Zhang, Pai Zheng, Mingxiao Yang, Yin Huang, E. Liu, Aonan Liu, Hui Zhang, Jing Guo

**Affiliations:** 1 Department of Dermatology, Chengdu University of Traditional Chinese Medicine, Chengdu, 6610075 People’s Republic of China; 2 Department of Dermatology, Hospital of Chengdu University of Traditional Chinese Medicine, No. 39 Shi-er-qiao Road, Chengdu, Sichuan Province 6610072 People’s Republic of China

**Keywords:** Microplastics, Gut–skin axis, Skin diseases, Microbial

## Abstract

Microplastics (MPs), ubiquitous environmental pollutants, can enter the human body through ingestion, inhalation, and dermal contact, accumulate in various organs, and exert harmful effects. Emerging evidence suggests that both the skin and the gut serve as key immunological and neuroendocrine organs, sharing structural and neuroanatomical similarities. The interaction between these two systems is referred to as the “gut–skin axis.” Numerous studies have demonstrated that MPs not only induce gut microbiota dysbiosis and compromise intestinal barrier integrity but also impair skin barrier function. Thus, the gut–skin axis offers a novel perspective for understanding MP-induced toxicity. Although interactions between MPs and the gut–skin axis have garnered increasing scientific interest, the mechanistic understanding of how MPs may mediate crosstalk between the gut and skin remains limited, and the impact of MPs on skin damage is not yet fully elucidated. MPs can directly disrupt gut microbial homeostasis and epithelial barrier function, allowing harmful bacteria and microbial metabolites to translocate into the bloodstream and exert systemic effects, ultimately contributing to cutaneous inflammation, metabolic imbalance, and oxidative stress. This review summarizes the mechanisms by which MPs exposure induces gut microbiota dysbiosis and skin damage from an integrated gut–skin axis perspective, highlighting their interplay’s relevance. Understanding changes in gut microbiota and its metabolites may represent a promising approach to mitigate MP-induced skin diseases via modulation of the gut–skin axis.

**Graphical Abstract:**

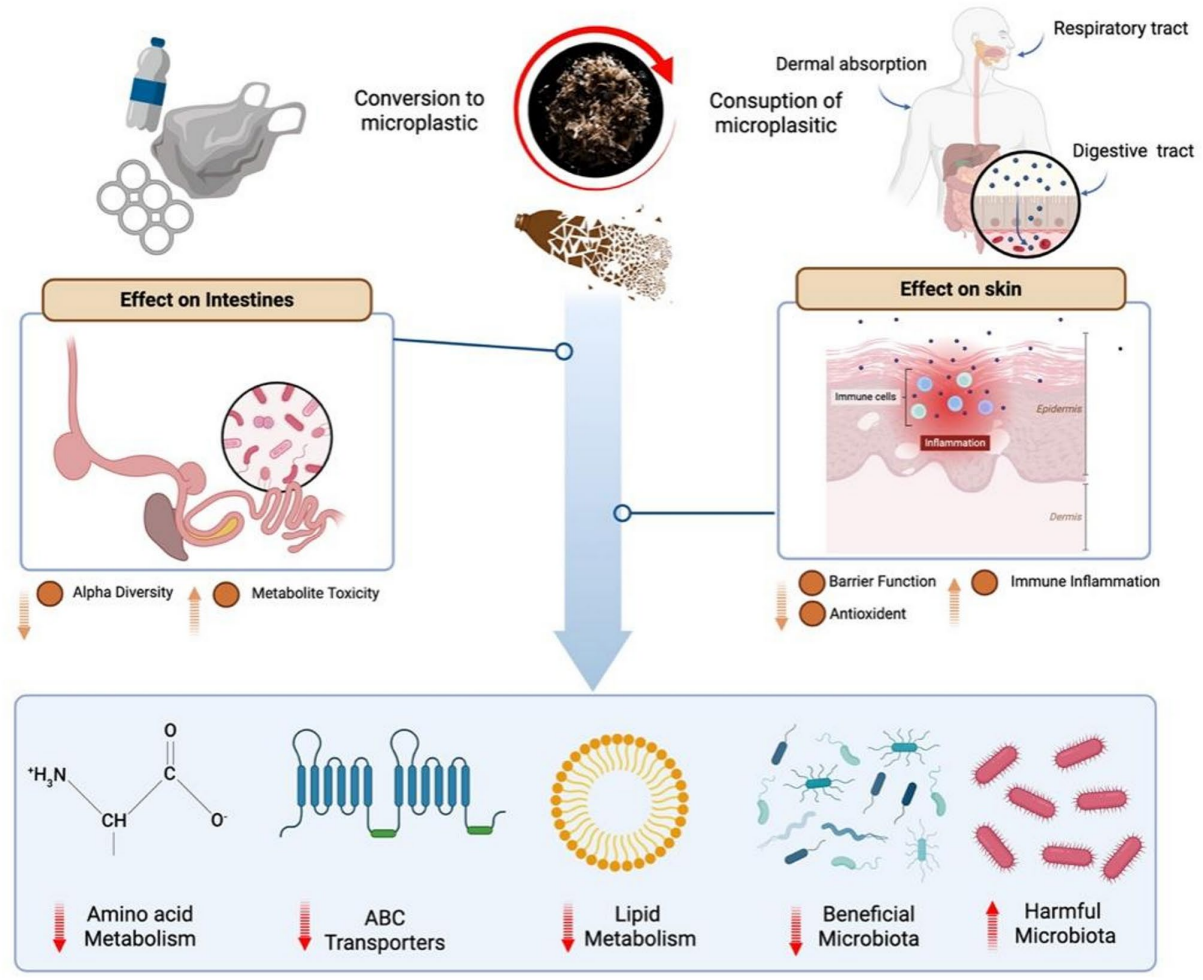

**Supplementary Information:**

The online version contains supplementary material available at 10.1186/s12967-025-07300-w.

## Highlights

Microplastics (MPs) compromise gut and skin barrier integrity through physical disruption and immune activation.

MPs trigger gut microbiota dysbiosis, leading to reduced SCFAs and tryptophan metabolites, thereby promoting chronic inflammation.

The gut–skin axis serves as a central mediator of MPs-induced dermatotoxicity via systemic immune crosstalk.

MPs activate innate and adaptive immune pathways, notably TLR2–MyD88–NF-κB and PPARγ signaling cascades.

Dysregulated metabolites impair antimicrobial peptide secretion and skin commensal balance, facilitating pathogen overgrowth (e.g., Staphylococcus aureus).

Modulation of gut microbiota and restoration of metabolic pathways may provide novel therapeutic strategies against MPs-induced skin injury.

**Supplementary Information:**

The online version contains supplementary material available at 10.1186/s12967-025-07300-w.

## Overview of microplastic and nanoplastic particles

Plastic has become one of the most extensively utilized materials in modern society, owing to its lightweight nature, chemical stability against acids and bases, and ease of molding into diverse forms. It finds widespread applications across sectors such as packaging, plumbing, and construction materials [1]. Since the mid-20th century, global plastic production has surged dramatically—from 2.3 million metric tons in 1950 to approximately 448 million metric tons by 2015. An estimated 8 million tons of plastic waste are discharged into marine environments annually by coastal regions [2]. Once introduced into ecosystems, plastic litter undergoes fragmentation through physical, chemical, and biological processes, giving rise to MPs—a term introduced by Thompson et al. in 2004 to describe plastic particles smaller than 5 mm in diameter [3, 4]. MPs have been widely identified not only in aquatic systems, including oceans and rivers, but also in terrestrial soils and the atmosphere. In fact, microplastic contamination on land may exceed levels in marine environments by factors ranging from four to twenty-three [5]. More recently, the detection of airborne MPs has prompted a shift in research focus toward terrestrial organisms, including vegetation, wildlife, and humans [6]. MPs pollution has been identified as a potential threat to ecosystems and biodiversity, and its adverse effects on human health have become a global public health concerns [5]. Due to their environmental persistence, bioaccumulative properties, widespread distribution, and high exposure potential, MPs have attracted increasing attention in the biomedical field, particularly in relation to tissue and organ damage, oxidative stress, inflammation, and neurotoxicity.

Recent findings suggest that MPs may negatively influence skin physiology. Experimental studies have demonstrated that MPs can compromise the integrity of the cutaneous epithelial barrier, promote oxidative stress, and activate immune-mediated inflammatory responses [7]. Therefore, elucidating the potential impact of MPs on skin integrity and human health is imperative. This review summarizes current concerns regarding the harmful effects of MPs on the skin, as reported in recent national and international literature. It also discusses mechanistic studies on MPs exposure and its effects on the gut microbiota and skin immune-inflammatory responses. Furthermore, the review proposes potential hypotheses linking MPs to the development of inflammatory skin diseases. These insights may provide a basis for further investigation into the toxicological mechanisms of MPs exposure on the “gut-skin axis,” the identification of potential biomarkers, and the development of bioprotective strategies to prevent.

## Methodology

This review employed a comprehensive search strategy across multiple academic databases, including PubMed, Web of Science, and Google Scholar [8]. Specific keywords and search strings were applied during the retrieval process, including *“Plastic”* [MeSH], *“Microplastics”*, *“Nanoplastics”*, *“Particles”*, *“Skin Diseases”* [MeSH], *“Gastrointestinal Microbiome”* [MeSH], *“Humans”* [MeSH], and *“Exposure Routes”*.The most recent search was conducted in March 2025 to ensure the inclusion of the latest available information. In addition, the reference lists of relevant publications were manually screened to identify any potentially overlooked studies pertinent to the topic. To ensure the inclusion of high-quality and relevant research, specific eligibility criteria were applied: (1) studies that investigated the detrimental impacts of MPs on the gastrointestinal and/or integumentary systems, especially those addressing immune and inflammatory responses; (2) research providing detailed characterizations of MPs physicochemical properties; (3) studies with well-defined methodologies, excluding those with insufficient data or poor-quality animal models; (4) literature relevant to biomedical or public health domains; and (5) publications that underwent peer review.

## Microplastics and barrier function: exposure pathways, physicochemical properties, and potential confounding factors

### Skin exposure and intestinal absorption pathways mediated by nanoplastics and microplastics

The skin, as the largest barrier organ in the human body, serves as a major entry point for microplastics (MPs). Humans are exposed to MPs through various routes, including environmental air, daily-use products, and personal care items. For example, certain cosmetics and skincare products contain micro/nanoplastic particles, such as polystyrene, which are used for scrubbing or exfoliation. Studies have shown that nanoplastics in personal care products typically range from tens of nanometers (e.g., 24 ± 6 nm to 52 ± 14 nm), theoretically allowing them to penetrate the skin and enter the body [9]. Research has demonstrated that when nanoplastics are carried by cosmetic carriers (such as oleic acid or ethanol), they can enhance transdermal penetration by dissolving stratum corneum lipids or increasing lipid mobility in intercellular spaces [10, 11]. The primary pathways for MPs to penetrate the skin are through the epidermis and skin appendages. The epidermal route involves particles reaching the dermis through intercellular spaces or within cells of the stratum corneum, from where they can be absorbed into the bloodstream via capillaries. Given the highly impermeable nature of stratum corneum cells, nanoplastics must undergo multiple hydrophilic/hydrophobic exchanges across cell membranes, resulting in minimal transcellular transport [12]. In contrast, smaller nanoplastics are more likely to penetrate the active epidermal layers via intercellular lipid bilayers. Meanwhile, the skin appendage route (e.g., hair follicles, sebaceous glands, sweat glands, and damaged skin) provides a relatively wide pathway for larger MPs. Studies suggest that particles up to several hundred nanometers in size can reach active layers beneath the epidermis through hair follicle channels [13].

An increasing body of epidemiological evidence indicates that MPs can enter the body not only via relatively wide cutaneous routes such as hair follicles and sweat glands, but also—under realistic exposure conditions—by penetrating the gastrointestinal barrier and reaching the circulatory system [13, 14]. It is estimated that, on average, individuals ingest about 40,000–50,000 microplastic particles annually through food, an amount comparable to what they inhale. Most of these particles remain in the intestinal lumen or respiratory tract and are eliminated through feces or mucus. However, approximately 2–3% of submicron particles can cross mucosal barriers—by active or passive transport, macrophage uptake, or transcellular routes—and enter the lymphatic and blood systems [15]. Clinical samples have detected MPs in blood, urine, thrombi, and placental tissues, with particle sizes predominantly ranging from 2.1 to 26 μm [16]. Reports of plastic polymers in neonatal umbilical cord blood, placenta, and meconium further suggest that MPs can traverse the placental barrier and enter the fetal gastrointestinal tract via the bloodstream [17]. Collectively, these findings demonstrate that under real-world exposure conditions, MPs can cross either the skin or gastrointestinal barriers and reach systemic circulation and other organs.

### The impact of physicochemical properties of microplastics on barrier permeability and toxicological effects

The size, shape, and surface chemistry of MPs significantly influence their interactions with biological barriers and subsequent toxicological effects.

(1) Size: Smaller particle sizes (especially at the nanometer scale) and larger specific surface areas facilitate easier penetration through the skin and gastrointestinal barriers, allowing for extensive interactions with cellular components, which may trigger stronger immune activation and cytotoxicity [16]. Experimental evidence indicates that only extremely small nanoparticles (e.g., < 45 nm) can penetrate intact skin, while nanoparticles up to several tens of nanometers in size may reach deeper dermal layers in cases of damaged or open skin [12]. For instance, in the lungs, particles smaller than 1 μm can penetrate the thin alveolar fluid layer and enter the bloodstream through active or passive cellular transport. Particle size and surface charge determine the ease with which particles are cleared by immune cells; positively charged or protein-coated particles are more readily bound to cell membranes and phagocytosed by macrophages [18]. In contrast, microparticles (>1 μm) are usually retained in the mucous layer or transported by M cells, exhibiting lower bioavailability. However, their larger size may cause physical friction and mechanical damage to tight junction proteins [19]. (2) Shape: The shape of the particles also significantly affects their distribution and toxicity within the body. Animal studies have shown that fibrous or plate-like MPs, with a higher aspect ratio and mechanical rigidity, are more likely to embed into the mucous layer, leading to intestinal mucosal damage, inflammation, and dysbiosis [20]. In contrast, microplastic fragments or beads exhibit lower toxicity. Environmental monitoring has also reported that fibrous MPs accumulate the most in the intestines, and exposure may exacerbate intestinal inflammation and microbial shifts [21]. Spherical particles primarily enter cells through endocytosis, and intracellular accumulation can result in lysosomal damage and disruption of autophagic flux [19]. (3) Surface Chemistry: The surface chemistry of MPs plays a critical role in their biological affinity and immune response. Studies have shown that particles with polar functional groups, such as carboxyl or amino groups, more readily bind to regions of cell membranes with opposite charges and are internalized through phagocytosis [22, 23]. When exposed to negatively charged carboxylated polystyrene particles (40 nm and 200 nm), human epithelial cells internalize them via phagocytosis and macropinocytosis [24].

### Potential confounding factors in the relationship between microplastics, microbiota, and skin health

**Fig. 1:**
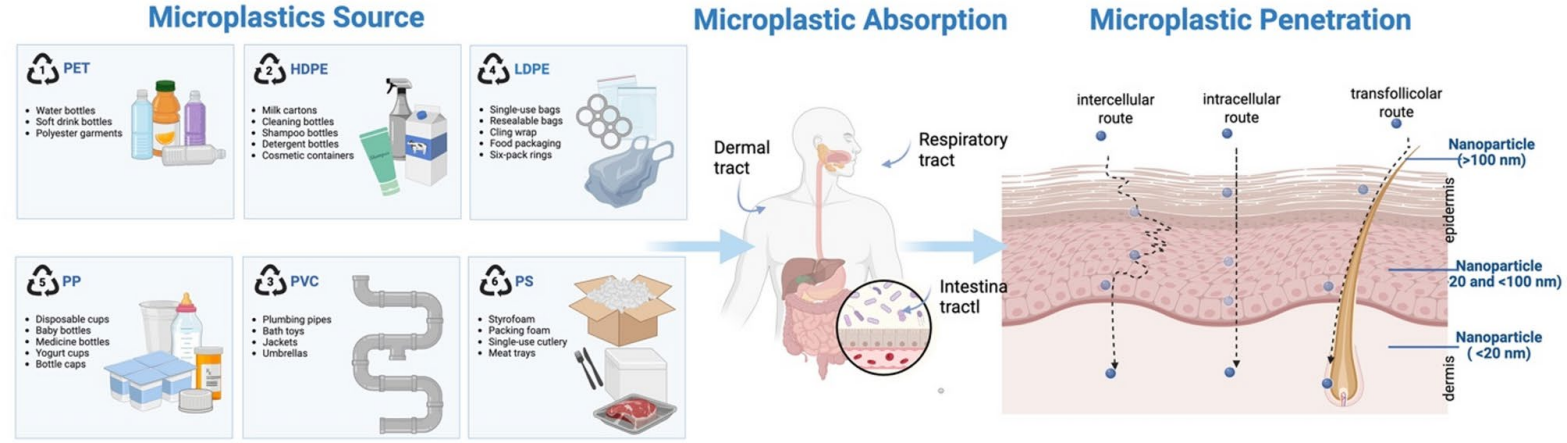
Microplastics and barrier disruption: exposure pathways and penetration. Polystyrene particles with diameters ranging from 20 to 200 nm can penetrate 2–3 micrometers into the superficial layers of the skin. NPs smaller than 100 nm can penetrate the stratum corneum via intercellular routes. In comparison, particles up to 200 nm can enter through skin furrows, lipid channels, and hair follicles, accumulating beneath the stratum corneum in the viable epidermis and even within cells

The impact of MPs on the gut-skin axis and microbiota is regulated by various external factors, with diet, genetic background, and co-exposure acting as key confounding variables. (1) Diet: Diet is one of the primary routes through which humans ingest MPs, with global estimates suggesting an annual intake of 39,000 to 52,000 particles per person. Diet al.so significantly influences gut microbiota composition [25]. Studies have shown that maternal dietary habits are correlated with microplastic levels in fetal meconium, and the types of plastic polymers found in fecal samples from different populations correspond to local dietary patterns and packaging practices [26]. (2) Host Genetic Background: The genetic background of the host plays an important role in maintaining barrier integrity and microbiota homeostasis. Although diet and environmental factors are major determinants of microbiota composition, host genetic polymorphisms still significantly impact microbiota structure and mucosal immune responses [27]. For example, mutations in skin barrier-related genes (such as filaggrin) can lead to increased transdermal water loss and downregulated expression of tight junction proteins, thereby enhancing the skin’s permeability to MPs. Variants in gut immunity-related genes (such as NOD2) may indirectly regulate the intestinal toxicity of MPs by altering microbiota composition and modulating the intensity of inflammatory signals. (3) Co-exposure: Co-exposure is another critical factor that exacerbates the toxicity of MPs. MPs can adsorb heavy metals (such as copper, arsenic, and lead) and persistent organic pollutants, and their ability to adsorb these pollutants is further enhanced after environmental aging. This combined exposure can lead to oxidative stress in the intestinal epithelium, barrier damage, and exacerbated inflammatory responses [28]. Additionally, MPs can serve as vectors for pathogenic microorganisms (such as Helicobacter pylori and SARS-CoV-2), increasing the risk of infection [29]. Therefore, when assessing the health risks of MPs, it is essential to consider the combined effects of an individual’s initial microbiota state, dietary structure, genetic susceptibility, and co-exposure to environmental factors (Fig. 1).

## Potential effects of MPs exposure on the gut and skin

### Nanoplastic-induced skin barrier disruption and its association with the immune system

In recent years, growing emphasis has been placed on the complex relationship between epithelial barrier integrity and inflammatory processes. Epithelial cells, as central components of innate immunity, are pivotal in maintaining tissue equilibrium. Their roles include enhancing mucociliary clearance, synthesizing antimicrobial peptides and pro-inflammatory mediators, activating local mucosal immune cells, and orchestrating the recruitment of immune cells to inflammatory or infected regions. Collectively, these functions establish a multifaceted frontline defense encompassing physical, chemical, and immune mechanisms [30]. Additionally, epithelial cells contribute to tissue regeneration and structural remodeling through rapid proliferation, directed migration, cellular differentiation, secretion of trophic factors, and enzymatic breakdown of extracellular matrix components [31]. The “epithelial barrier theory” posits that environmental toxicants may impair the protective capabilities of the skin and mucosal barriers. Due to their nanoscale dimensions and structural similarity to subcellular organelles, nanomaterials are particularly prone to biological interaction and cellular uptake [32, 33]. MPs, including NPs, can serve as carriers for active or toxic molecules that may penetrate the skin, thereby increasing their potential health risks [34]. For instance, cosmetic-derived NPs have been found to exert cytotoxic effects on human keratinocytes by initiating oxidative stress responses, inhibiting cell division, and accelerating the onset of cellular aging [35].

Research has demonstrated that airborne nanoplastics, such as 40 nm polystyrene particles, can induce oxidative stress and inflammatory signaling, leading to cellular damage and disruption of epithelial integrity. These effects are closely related to both particle size and exposure duration [36]. Although data on the skin penetration capacity of NPs remain limited, studies have shown that polystyrene particles ranging from 20 to 200 nm in diameter can infiltrate up to 2–3 microns beneath the skin surface [37]. Particles smaller than 100 nm can penetrate the stratum corneum via the intercellular lipid matrix, while particles up to 200 nm may gain entry through skin furrows, lipid channels, and hair follicles, eventually accumulating in the viable epidermis or even entering cells [38, 39]. Thus, the health risks associated with transdermal NP penetration should not be overlooked.

Exposure to MPs and NPs—whether acute or chronic—can initiate oxidative stress, leading to the activation of cellular autophagy and apoptotic pathways, thereby contributing to dermatological disorders such as atopic dermatitis, premature skin aging, and alopecia [40]. Upon dermal contact, these particles are detected by pattern recognition receptors (PRRs) on a range of skin-resident cells, including keratinocytes, fibroblasts, dendritic cells, melanocytes, macrophages, and T lymphocytes. This detection prompts the production of antimicrobial peptides and pro-inflammatory cytokines (e.g., IL-1, IL-6, IL-10, TNF-α), immune cell infiltration, and the upregulation of apoptosis-related proteins such as BAX, caspase-3, and caspase-8, which collectively impair skin barrier functionality through mitochondrial-mediated apoptosis [41]. Furthermore, MPs/NPs can be internalized by cells via various endocytic mechanisms, including phagocytosis, macropinocytosis, and clathrin- or caveolin-dependent pathways, leading to intracellular buildup within cytoplasmic and lysosomal compartments [42]. Such accumulation results in direct cytotoxicity driven by inflammation and oxidative stress [43]. Additionally, pre-existing conditions such as skin inflammation, allergic responses, or aging can enhance the permeability and internalization of MPs/NPs, increasing susceptibility to their toxic effects [44] (Table 1).

**Table 1:** Effects of Microplastics/Nanoplastics on skin tissue and the underlying mechanisms

Model & cell type	MPs type & diameter	Exposure concentration	Exposure duration	Target cell type	Mechanism	Reference
C57BL/6J mice; fibroblasts	Polystyrene (PS), 150 μm	0, 50, 100, 200, 400 µg/mL	24 h	Fibroblasts	Inhibits COL1A1, COL1A2, SPP1 expression, disrupting ECM homeostasis and accelerating skin aging.	[45]
HaCaT cells; C57BL/6 mice	PS, 100 nm	10, 50, 100 µg/mL (cells); 12.5, 25 mg/kg (mice)	24–72 h (cells); 18 days (mice)	HaCaT keratinocytes	Induces mitochondrial oxidative stress, GSDMD-mediated mtDNA release, activating AIM2 inflammasome and triggering inflammation and cellular senescence.	[46]
HaCaT, SCL-1, A431 cells	Polyethylene (PE), 1 μm	0.25, 0.5, 1 mg/mL	24 h	HaCaT and SCC cells	Increases mitochondrial ROS and membrane potential changes, activating NLRP3 via mtDNA leakage and promoting proliferation of skin cancer cells.	[47]
Dermal fibroblasts and keratinocytes from SKH1-hr mice	Fluorocarbon (FB) PS, 200 nm–6 μm	100 µg/mL	24 h	Fibroblasts and keratinocytes	Enhances intracellular ROS, activates Nrf2-dependent antioxidant response, and alters β-catenin signaling.	[48]
C57BL/6 mice; HaCaT, L929 cells	Aged PS, 120 nm	30 mg/kg (mice); 24 h (cells); 60 days (mice)	24 h (cells). 60 days (mice)	HaCaT and L929 cells	Exacerbates toxicity in skin and follicular cells, induces mitochondrial apoptosis, disrupts follicle-shaft adhesion, leading to hair loss.	[49]

### Nanoplastic-induced intestinal barrier disruption and its association with the immune system

Among various exposure pathways, oral intake is recognized as the dominant route through which humans encounter MPs. These particles may enter the digestive system through direct ingestion or indirectly via polluted food items, beverages, packaging materials, and containers made of plastic, glass, or paper [50]. Evidence indicates that ingested MPs accumulate within the gastrointestinal tracts of various species [21, 51]. For example, oral administration of 60 nm polystyrene (PS) nanoparticles in rats resulted in approximately 10% of the administered dose remaining in the GI tract [52]. A review by the European Food Safety [53]: MPs exceeding 150 μm in diameter are generally not absorbed by the human body but instead remain adherent to the mucus layer, maintaining close contact with the apical membranes of intestinal epithelial cells. Conversely, particles smaller than 150 μm may penetrate the mucus barrier. Four key mechanisms have been proposed to explain the size-dependent intestinal uptake of nano- and microparticles: (1) endocytosis by enterocytes; (2) transcytosis mediated by M cells within gut-associated lymphoid tissues; (3) persorption, where particles cross the epithelium at villus tips following epithelial cell extrusion; and (4) paracellular diffusion [54]. Owing to their small size, NPs can reach various organs, resulting in systemic toxicological exposure. Experimental evidence suggests that once internalized, NPs may localize in the liver, spleen, lungs, heart, skin, reproductive organs, and even traverse the blood–brain barrier to reach neural tissues [55].

MPs exposure may directly damage the gut mucosa, disrupt mechanical barrier integrity, and alter intestinal permeability and microbiota composition [56, 57]. These disruptions are associated with oxidative stress, DNA damage, inflammation, genotoxicity, cell membrane injury, and apoptosis, all of which contribute to the breakdown of intestinal barrier function [58, 59]. Experimental studies demonstrate that MPs elevate intestinal and plasma concentrations of diamine oxidase (DAO) and D-lactate (D-Lac), while suppressing the expression of tight junction proteins (e.g., claudins, ZO-1, and occludin) [60].

Barrier disruption enables MPs to translocate into systemic circulation, exacerbating their toxicological impact [61]. For example, polyethylene microplastics (PE-MPs) promote blood cell apoptosis, dysregulate phagocytic activity, and impair innate immune homeostasis [62]. Enzymes such as acid phosphatase (ACP), alkaline phosphatase (AKP), and lysozyme (LZY) are representative of innate immune activity and often serve as biomarkers of physiological stress or disease. During exposure to MPs and NPs, these immune markers exhibit a biphasic, concentration-dependent response: their levels initially increase but subsequently decline upon prolonged or high-dose exposure, indicating that excessive stress may lead to immune exhaustion or suppression.

### Microplastic-induced gut microbiota dysbiosis and immune-inflammatory damage

In summary, MPs disrupt host immune regulation through multiple mechanisms, including aberrant activation of pattern recognition receptors, complement dysregulation, apoptosis, redox imbalance, and lipid metabolic reprogramming. The resulting immune dysregul. The gut microbiota is critical for maintaining mucosal barrier integrity and regulating host immunity. Oral ingestion of MPs weakens the intestinal mucosal barrier, leading to bacterial translocation and dysbiosis [63–66]. Animal studies show that MPs exposure induces structural damage to intestinal villi, reduces crypt numbers, downregulates tight-junction proteins such as ZO‑1 and Occludin, and thins the mucus layer, resulting in increased intestinal permeability [67]. This compromised barrier allows intestinal microorganisms and MPs themselves to migrate into the submucosa and circulation, triggering bacterial translocation and systemic inflammatory responses. Moreover, MPs provide attachment surfaces that promote biofilm formation, alter the local microbiome, and affect microbial colonization [68, 69]. Some intestinal bacteria can metabolize additives on MPs (e.g., plasticizers), releasing toxic chemicals and further exacerbating mucosal injury and microbial imbalance [70]. In vitro digestion models and artificial gut systems have clarified how MPs influence microbiota composition. During simulated human gastrointestinal digestion, polyethylene microparticles significantly increased the relative abundance of pathogenic bacteria, including *Clostridium*,* Bacteroides*, and *Escherichia* [71]. In fecal microbiota incubation models, MPs caused excessive proliferation of the phylum *Proteobacteria* (e.g., *Desulfovibrio* and *Enterobacteriaceae*) while inhibiting beneficial taxa from the phylum Firmicutes, such as *Lactobacillaceae* and *Bifidobacteriaceae* [72]. Biodegradable plastics (e.g., polycaprolactone (PCL) and polylactic acid (PLA)) similarly reduced microbial α-diversity in colonic fermentation, lowering the abundance of beneficial bacteria such as Lactobacillus and Bifidobacterium. These compositional shifts are typically accompanied by decreased short-chain fatty acid synthesis and abnormal bile acid metabolism, which may indirectly mediate MPs’ toxic effects on distant organs—including the liver and brain—through low-grade inflammation and metabolic disturbances [67]. In vivo animal studies corroborate MPs-induced dysbiosis and its physiological consequences; numerous fish and rodent models have shown that long-term MPs exposure disrupts gut microbiota structure and amplifies local inflammation [73]. For instance, mice on a high-fat diet exposed to polystyrene MPs exhibited enrichment of *Desulfovibrio* and *Clostridium* and a marked reduction in Lactobacillus and Bifidobacterium [74].

Human observational studies also implicate MPs in altering the gut microbiota. Epidemiological surveys indicate that individuals who frequently consume plastic-packaged foods show significant shifts in the relative abundances of several phyla—including *Actinobacteria*,* Proteobacteria*,* Firmicutes*,* and Bacteroidetes*—with a notable increase in the gas-producing genus Collinsella [75]. MPs can cross the placenta, affecting the fetus. MPs, consisting mainly of polyamide (PA) and polyurethane (PU), have been detected in placental tissues, with concurrent changes in β-diversity and bacterial abundance in fetal meconium [76]. In infant populations, bottle-fed babies exhibit reduced fecal microbiota diversity and decreased numbers of beneficial bacteria such as *Bifidobacterium and Parabacteroides* [77]. In occupationally exposed groups like plastic production workers, abnormal fecal microbiota profiles have been observed, including increased *Bifidobacteriaceae and Streptococcus* with reduced levels of beneficial genera like *Ruminococcus and Dorea* [78]. Notably, individuals with inflammatory bowel disease (IBD) have significantly higher MP concentrations in their feces than healthy controls, and these concentrations correlate with disease severity, suggesting that MPs may exacerbate intestinal inflammation by worsening microbial imbalance [79].

At the immunological level, MPs disrupt homeostasis through several interlinked molecular pathways, precipitating inflammatory responses, oxidative stress, and apoptosis, ultimately leading to immune dysfunction and related diseases. MPs are recognized by the immune system either as particulate foreign bodies or via their low-molecular-weight soluble components (e.g., plasticizers, bisphenols), which mimic damage-associated molecular patterns (DAMPs). This recognition activates membrane-bound Toll-like receptors (TLRs), particularly TLR2 and TLR4 [80]. Downstream signaling through IRAK and TRAF6 via the MyD88 adaptor protein activates the IKK/NF‑κB pathway, promoting the release of pro-inflammatory cytokines such as TNF‑α, IL‑1β, and IL‑6 and increasing reactive oxygen species (ROS), thereby generating a localized “inflammatory storm.” [81] In vivo studies have shown that MPs exposure upregulates TLR4, MyD88, and phosphorylated NF‑κB, while inhibitors of TLR2/4 markedly reduce inflammatory damage, highlighting the importance of this pathway in MPs-induced immune toxicity [82]. Excessive ROS not only causes direct cellular damage but also triggers NLRP3 inflammasome activation, promoting caspase‑1 activation and the maturation and release of IL‑1β and IL‑18. This feedback loop of “oxidative stress–inflammation” further induces programmed cell death (apoptosis and pyroptosis) and disrupts the Th1/Th2 immune balance [83].

In the complement system and innate immunity, MPs exposure causes a transient upregulation of complement components (e.g., C3 and C4) followed by a decline under prolonged or high-dose exposure, illustrating nonlinear dynamics. MPs may inhibit the formation of the membrane attack complex (MAC) and disrupt the C3a/C5a signaling axis, suggesting that they modulate complement function through both inflammatory pathways and microbial dysbiosis [84]. Such disturbances impair antigen clearance and mucosal barrier maintenance, increasing susceptibility to infection and local inflammation. Concerning antimicrobial enzymes and phagocytic function, the effects of MPs on LYZ and phenoloxidase (PPO) activities are model-dependent: short-term or low-dose exposure may upregulate LYZ expression and activity, possibly as a compensatory response, whereas other contexts show enzyme inhibition or dysfunction, reflecting the complexity of MPs–microbiota–host interactions [85].

MPs also indirectly affect mucosal immune homeostasis and polymeric immunoglobulin receptor (pIgR) expression by disrupting gut microbiota composition. pIgR mediates the transport of secretory IgA and IgM and is key to maintaining the intestinal mucosal barrier [86–88]. Its expression correlates positively with beneficial bacteria such as Bacteroides and members of Actinobacteria and is tightly regulated by cytokines (e.g., TNF‑α, IFN‑γ, IL‑4, TGF‑β) and chemokines (e.g., CXCL12/CXCR4) [89]. MPs-induced dysbiosis and local inflammation can downregulate pIgR, disrupt lymphocyte homing and antigen presentation, and weaken mucosal defenses. At the metabolic level, MPs interfere with nuclear receptor PPAR signaling (PPARα/β/γ), causing lipid metabolism disorders, mitochondrial dysfunction, and energy metabolism abnormalities. These effects diminish the metabolic flexibility and effector functions of immune cells (e.g., macrophages and T cells), making them more susceptible to dysfunction and intensifying immune damage during chronic inflammation [90] (Refer to Supplementary Table 1 for details).

Collectively, experimental findings reveal a causal link between MPs and the disruption of host immune–metabolic homeostasis. MPs drive immune–metabolic imbalance through multiple mechanisms: (1) direct or indirect activation of the TLR–MyD88–NF‑κB pathway via their surface properties or soluble components; (2) induction of ROS production and NLRP3 inflammasome activation; (3) interference with complement and antimicrobial enzyme functions; (4) modulation of pIgR expression and mucosal barrier function through the microbiota–immune axis; (5) reprogramming of lipid metabolism and mitochondrial function; and (6) transport or adsorption of heavy metals, persistent organic pollutants, and pathogens, with weathered “eco-coronas” that enhance their interaction with immune receptors. These mechanisms converge to form a multidimensional network that promotes inflammatory imbalance, immune suppression, and metabolic disorders, providing molecular and cellular insights into the association between environmental MP exposure and a range of immune-related diseases.

## Gut microbiota-mediated skin damage

### Gut microbiota-mediated skin damage via the gut–skin axis

In the early 20th century, dermatologists Stokes and Pillsbury first proposed the concept of communication between the gut, skin, and brain [91]. Both the gut and the skin are exposed to the external environment and host diverse microbial communities [92]. Circulating microbes act as messengers within the gut–skin axis, primarily via immunological pathways influenced by diet, stress, and environmental factors, which regulate intestinal and skin barrier homeostasis [93, 94].The immune system regulates host–microbiota interactions, with microbiota impacting autoimmune and immune-mediated processes via molecular mimicry, bacterial translocation after barrier disruption, modulation of immune cells in Peyer’s patches, and production of immunomodulatory metabolites [95, 96]. The gut mucosal immune system features a multilayered “firewall” of epithelial cells, mucus, secretory IgA, and immune cells (dendritic cells, T lymphocytes), limiting microbial access to gut-associated lymphoid tissue (GALT) and preventing systemic immune activation [97–99]. Certain gut bacteria, including *Bacteroides*, *Bifidobacterium*, *Prevotella*, *Faecalibacterium*, *Lactobacillus*, and *Parabacteroides*, ferment indigestible polysaccharides into short-chain fatty acids (SCFAs)—such as butyrate, acetate, and propionate—which enhance epithelial barrier integrity and reduce gut permeability, thereby preventing microbial translocation to extraintestinal tissues [100, 101]. Other metabolites, including trimethylamine (TMA), trimethylamine N-oxide (TMAO), secondary bile acids, and tryptophan derivatives, are also crucial for immune homeostasis and systemic organ function [100].

Microbial dysbiosis—characterized by diminished microbial diversity and altered community composition—has been implicated in the disruption of epidermal differentiation signals, modulation of immune function, activation of proinflammatory pathways, and compromise of skin barrier integrity through microbe-derived metabolites [93]. These disturbances are closely linked to the onset and progression of dermatological conditions such as acne, atopic dermatitis (AD), psoriasis, and rosacea [102–104] (Refer to Supplementary Table 2 for details). As discussed in previous sections, MPs exposure may increase the abundance of harmful bacteria (e.g., *Desulfovibrio*, *Erysipelothrix*, *Helicobacter*, *Alistipes*, *Rhodobacteraceae*) while decreasing levels of beneficial commensals (e.g., *Lactobacillus*, *Bacteroides*, *Akkermansia*) [105]. Among these, increased *Bacteroidetes* and *Proteobacteria* and decreased *Firmicutes* are the most prominent changes, often accompanied by disruptions in lipid metabolism, SCFA synthesis, and protein digestion and absorption. These alterations compromise epithelial barrier integrity, elevate intestinal permeability, and promote the development of “leaky gut syndrome,” which in turn triggers inflammatory responses in both the gut and skin [106, 107]. Intestinal-derived molecules—such as SCFAs and neurotransmitters including γ-aminobutyric acid (GABA), acetylcholine, dopamine, and serotonin—may enter the bloodstream and interact with TLR-expressing cells within the skin. This interaction can induce the secretion of proinflammatory cytokines such as interleukin-12 (IL-12) and interferon-gamma (IFN-γ), which in turn modulate skin-resident microbial communities and trigger systemic inflammatory cascades [106, 108].

### The gut–skin axis perspective: gut microbiota as a mediator between microplastics and skin interactions

Although numerous studies have demonstrated the toxic effects of MPs on both the gut and skin, evidence for gut microbiota or its metabolites mediating MP-induced skin damage remains relatively limited. Following oral ingestion, MPs profoundly alter gut microbiota composition and function. Within the theoretical framework of the “gut–skin axis,” there is a strong association between the gut microbiota, its metabolites, and the development of skin diseases. MPs induce immune-related mechanisms such as the activation of recognition receptors, complement system function, apoptosis, redox homeostasis, and lipid metabolism. These disruptions trigger inflammation imbalance, immune suppression, and metabolic abnormalities, which may represent key potential mechanisms underlying various immune-related diseases and provide a theoretical basis for the association between environmental MPs exposure and skin immune system disorders.

As previously discussed, MPs exposure decreases the relative abundance of beneficial gut bacteria and increases opportunistic pathogens. These shifts correlate significantly with skin biochemical markers. Gut dysbiosis can impair barrier function and promote translocation of harmful substances, such as endotoxins, thereby contributing to skin damage. Specifically, changes in gut microbiota diversity can affect epidermal differentiation pathways, immune regulation, inflammatory signaling, and barrier integrity mediated by microbial metabolites, ultimately destabilising skin immune homeostasis [93]. Increasingly, evidence indicates that dysbiosis influences skin health through the “gut–skin axis.” The following microbial groups and metabolites are particularly important mediators of MPs’ effects along this axis.

*Ruminococcus gnavus(R. gnavus)*, a member of the Firmicutes phylum (family Ruminococcaceae), plays a key role in fiber degradation and the production of SCFAs, thereby influencing host metabolism and immune regulation. Research indicates that *R. gnavus* expresses immunoglobulin-binding proteins (IbpA/IbpB) on its cell surface, acting as a B cell superantigen [109]. These proteins bind to the variable region of IgA and induce a strong plasma cell response, leading to the dense coating of *R. gnavus* by IgA. Isolated strains of *R. gnavus* can produce capsular polysaccharides, which promote the secretion of inflammatory cytokines such as tumor necrosis factor-α (TNF-α) in dendritic cells via Toll-like receptor 4 (TLR4) [110]. These characteristics link *R. gnavus* to the activity of autoimmune or inflammatory diseases, such as systemic lupus erythematosus (SLE). Studies have found that the number of *R. gnavus* in the fecal samples of infants with AD is lower than in healthy controls and is negatively correlated with TLR2-induced IL-6 and TNF-α [111]. Azzouz et al. observed increased sIgA-coated *R. gnavus* in SLE patients, with proliferation directly proportional to disease activity [112]. This is because abnormal expansion of *R. gnavus* often coincides with impaired gut barrier function, elevated serum calprotectin, and higher lipopolysaccharide (LPS) levels, suggesting that it exacerbates disease by promoting inflammatory cytokine secretion and increasing gut permeability.

Several other Firmicutes bacteria, such as *Butyrivibrio*,* Bacillus*,* Lactococcus*,* Lactobacillus*,* Faecalibacterium*,* Clostridia*,* Anaerostipes*,* and Ruminococcus*, are believed to have anti-inflammatory effects. Their decreased abundance in acne patients correlates with increased inflammation [113]. *Butyrivibrio* produces butyrate, which provides energy to colonic cells, enhances barrier function, and suppresses inflammation [114]. *Clostridia* and *Faecalibacterium* regulate the gut-skin axis by producing SCFAs [115]. *Bacillus* species synthesise antimicrobial compounds such as 3‑hydroxypropionaldehyde along with common probiotics [116]. Declines in *Lactococcus*,* Bacillus*, and *Clostridia* numbers are associated with mTOR pathway suppression—a key driver of sebaceous gland proliferation and secretion [117]. *Lactobacillus* improves gut health through anti-inflammatory regulation, enhancing barrier integrity and reducing intestinal permeability. Its increased abundance in patients with IBD suggests a potential shared pathogenesis with rosacea [118]. Research has shown that in males, long-chain saturated fatty acids such as alpha-linolenic acid, linoleic acid, and stearic acid significantly increase, while the short-chain fatty acid valproic acid decreases [119]. Linoleic acid is a pro-inflammatory substance, and its elevation correlates with the downregulation of the PI3K/Akt/mTOR pathway, which contributes to the severity of acne vulgaris [120].

*Bacteroidetes* and *Firmicutes* together constitute the dominant phyla in the gut microbiota. *Bacteroides*, a representative genus, activates TLR2 and stimulates anti-inflammatory cytokine production (IL‑10, IL‑17, IL‑21) via polysaccharide A, maintaining immune balance in both gut and skin [121–123]. Dysbiosis caused by microplastic exposure typically manifests as fluctuations in the Firmicutes/Bacteroidetes (F/B) ratio, with an increase or decrease in this ratio being considered a potential marker for skin diseases such as alopecia areata, systemic lupus erythematosus (SLE), and psoriasis. For instance, *Bacteroides* abundance declines significantly in alopecia areata, while in SLE it correlates positively with serum IL‑2 levels [124, 125].Probiotics such as *Bifidobacterium* (phylum Actinobacteria) are also pivotal in the gut–skin axis. These microbes not only contribute to SCFA synthesis but also support skin health through antioxidant effects and gut-barrier reinforcement [126, 127]. Conversely, certain *Clostridium* species (phylum Firmicutes) are enriched in melanoma and colorectal cancer, where they promote tumour progression by activating the Wnt pathway and suppressing immune responses; this pathogenic expansion reflects microbial reshaping and heightened inflammation risk associated with MPs exposure [128, 129]. *Propionibacterium*, a Gram-negative anaerobe within the phylum Bacillota, is linked to reduced risks of ADand rosacea; its abundance is significantly lower in AD patients’ gut microbiota [130].

Microplastic exposure not only disrupts gut microbiota but also alters host immune balance by diminishing microbial metabolic capacity. In the gut–skin axis, key metabolites include SCFAs, tryptophan derivatives, and secondary bile acids. Connecting changes in gut composition to metabolic function offers insight into the mechanisms underpinning the gut–skin axis and MP-induced toxicity.

#### Short-chain fatty acids (SCFAs)

SCFAs are among the most extensively studied metabolites in host-microbiota interactions. They are a group of fatty acids produced by intestinal bacteria during the fermentation of dietary fibers. SCFAs are primarily produced by microbiota such as *Firmicutes and Bacteroidetes* and are transported via the bloodstream to the skin, where they bind to G-protein-coupled receptors (e.g., GPR41/43, GPR109A) and nuclear receptors like PPARγ on keratinocytes and immune cells. Their biological effects include enhancing regulatory T cell (Treg) function, promoting keratinocyte differentiation, and suppressing pro-inflammatory cytokine production by neutrophils and macrophages [131–134]. SCFAs also regulate gene expression by inhibiting histone deacetylases (HDACs), maintain the Th17/Treg balance, and stimulate Staphylococcus epidermidis to produce antimicrobial peptides that limit colonization by Staphylococcus aureus [135]. Low SCFA levels have been linked to an increased risk of ADin children; SCFAs modulate the FcεRI-mediated signaling cascade epigenetically, thereby inhibiting allergic responses such as mast cell degranulation [136, 137].

In psoriasis, SCFAs exert their effects through: (i) activation of GPCRs and inhibition of HDACs, (ii) regulation of both innate (neutrophils, macrophages, dendritic cells) and adaptive (T and B lymphocytes) immune responses, particularly correcting Th17/Treg imbalance, and (iii) maintenance of gut barrier integrity [138]. In alopecia areata patients, alterations in microbiota composition—including decreased Bacteroides and increased Firmicutes—may reflect SCFA-mediated effects on intestinal Tregs [139]. Tregs are crucial for peripheral tolerance and preventing autoimmune disease, and they are particularly abundant in hair follicles [140]. Microplastic exposure reduces SCFA-producing bacteria such as *Faecalibacterium and Akkermansia*, resulting in decreased SCFA synthesis, which contributes to gut barrier dysfunction and exacerbated skin inflammation.

##### Tryptophan metabolites

Tryptophan metabolites, produced by bacteria such as *Clostridium sporogenes*, *Bacteroides*, *Bifidobacterium*, and *Lactobacillus*, include indole-3-aldehyde and indole-3-propionic acid, which are natural ligands for the aryl hydrocarbon receptor (AHR) [141]. AHR activation in keratinocytes, Langerhans cells, and fibroblasts upregulates the expression of antimicrobial peptides, promotes the synthesis of filaggrin and loricrin, induces the release of IL-22 and prostaglandin E2, and inhibits the production of inflammatory cytokines [142–144]. Microbial-derived AHR ligands not only strengthen the epidermal barrier but also regulate the Th17/Treg balance. However, the dysbiosis caused by microplastics reduces the indole-producing microbiota, leading to insufficient tryptophan metabolites and disrupting this immune regulatory mechanism [145]. In the skin, AHR activation elicits diverse responses: in keratinocytes of AD patients, it upregulates filaggrin and loricrin to improve the skin barrier; in dermal fibroblasts, it enhances metalloproteinase production and suppresses type I collagen and fibronectin, thereby aiding wound healing and reducing scarring [146]; in macrophages, it decreases pro-inflammatory cytokine production, thereby inhibiting chemokine induced Th17 polarization [147]. *Bifidobacterium longum* can metabolize tryptophan into indole-3-aldehyde (I3C), which activates the AHR-mediated immune signaling pathway, inhibiting Th2 cells and alleviating AD [148].

#### Secondary bile acids

Secondary bile acids are generated by the gut microbiota through the conversion of primary bile acids, including lithocholic acid (LCA), deoxycholic acid (DCA), and its derivative 3-oxoLCA. These acids bind to nuclear receptor FXR and membrane receptor TGR5, downregulating inflammatory pathways such as NF-κB and NLRP3, and promoting the generation of regulatory T cells while inhibiting Th17 cell differentiation [149–151]. Additionally, they also modulate dendritic cell and macrophage polarization, maintaining immune homeostasis in both gut and skin [152]. However, microplastic-induced dysbiosis may reduce bile acid conversion efficiency, weakening their anti-inflammatory effects. Microplastic-induced dysbiosis may reduce bile acid conversion efficiency, weakening these anti-inflammatory actions. Evidence suggests that bile acids could have therapeutic potential in immune-mediated skin diseases: in an IL‑23 minicircle-induced psoriasiform dermatitis model, oral or intravenous administration of LCA, DCA, and 3-oxoLCA significantly decreased ear erythema and skin thickening, suppressed IL‑17 A production, and blocked CCL20‑CCR6-mediated T cell migration [153].

**Fig. 2:**
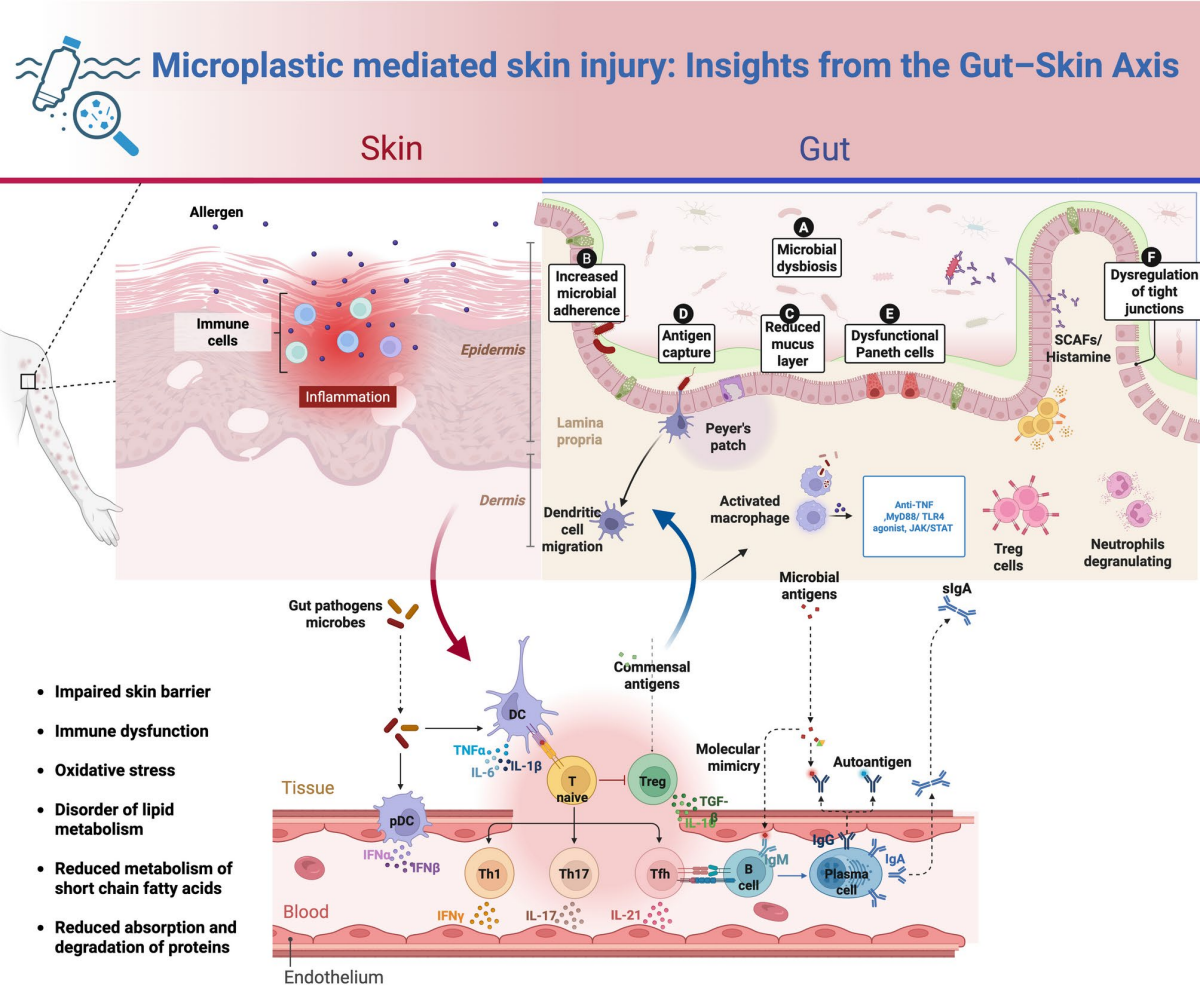
The connection between MPs and the gut–skin axis. Ingested MPs accumulate in the gut and are absorbed through intestinal epithelial cells via endocytosis, transcytosis, excessive adsorption, and paracellular uptake, thereby compromising the integrity of the intestinal mucosal barrier and altering the composition and function of the gut microbiota. These changes can lead to bacterial translocation and dysbiosis. Following direct intestinal injury, the reduced diversity of the gut microbiota influences epidermal differentiation signaling pathways, immune components, and inflammatory signaling, or disrupts barrier integrity via microbial metabolites, ultimately impairing skin immune homeostasis (e.g., causing inflammatory damage, metabolic dysfunction, and oxidative stress) and negatively impacting skin health

In summary, SCFAs, tryptophan metabolites, and secondary bile acids are central metabolites in the gut–skin axis, regulating immune responses and maintaining barrier integrity. Reduction of these metabolites constitutes an important mechanism by which microplastics exacerbate inflammatory skin diseases. Replenishing these metabolites or restoring the microbiota that produces them may offer promising avenues for future interventions (Fig. 2).

## Discussion and future directions

Despite growing awareness of MPs exposure and its effects on the human gut and skin, objective insights into their interactions remain limited. Although the mechanisms outlined above suggest a link between MPs-induced dysbiosis and skin health via the gut–skin axis, definitive evidence of causality is lacking. Moving forward, research should address the following key points:

**(1)** Currently, there is no animal model conclusively demonstrates that microbiota changes from oral MPs ingestion directly trigger dermatological conditions such as ADor psoriasis. Existing population studies are largely observational; for instance, higher microplastic content in nasal lavage fluids from allergic rhinitis patients than in healthy controls suggests correlation rather than causation [154]. Future research should prioritize large, well-controlled prospective cohort studies that carefully account for dietary and environmental confounders. Moreover, exposing animals to environmentally relevant MPs doses through oral or dermal routes, followed by fecal microbiota transplantation (FMT), could test whether skin inflammation is reproducible. Such experiments would clarify causal links and underlying mechanisms between MP-induced dysbiosis and skin inflammation.

**(2)** The “gut-microbiota-gut-skin axis” is not only a key pathway for the harmful effects of microplastic exposure but also a potential target for preventing and counteracting MP exposure risks. The gut barrier damage and microbiota dysbiosis induced by microplastics provide a theoretical basis for the application of probiotics, prebiotics, tight-junction modulators, and topical barrier protectants [155]. On the one hand, probiotics and prebiotics can restore microbiota diversity, enhance mucin and SCFA production, and lower endotoxin levels, thereby preserving tight-junction integrity and reducing intestinal permeability [156]. These effects substantially decrease systemic inflammatory markers such as TNF‑α and IL‑6 [157]. Engineered probiotics have also shown advantages in inflammatory models involving nanoplastics, including modulation of the microbiome, suppression of inflammation, and prevention of bacterial colonization [158, 159]. On the other hand, barrier protectants such as tight-junction-modulating peptides (e.g., Zonulin), mucosal immune modulators, or topical skincare products containing sebum components can help repair gut and skin barriers, reducing microplastic penetration and inflammation [160, 161]. Additionally, plant-derived polyphenols (e.g., citrus flavonoids like nobiletin, anthocyanin‑3‑O‑glucoside) and functional bioactive peptides have demonstrated protective effects in animal studies [162]. For example, anthocyanin‑3‑O‑glucoside promotes MPs excretion, normalizes gut microbiota, and restores barrier function in PS MP‑exposed mice [163]. By restoring barrier integrity, modulating immune microenvironments, and removing adsorbed pollutants, these interventions offer promising strategies to mitigate MPs toxicity. Future research should systematically evaluate their efficacy and safety in sterile animal models and FMT experiments under exposure conditions relevant to humans.

**(3)** It is important to note that most current experimental exposure conditions (such as high-dose, short-term ingestion) are not representative of typical human environmental exposure [71, 164]. When assessing MPs toxicity, exposure dose and duration are critical factors. Epidemiological data estimate that the average adult consumes dozens to hundreds of microplastic particles daily [165]. However, the exposure conditions in current studies differ significantly from real-world environments, with limitations such as using only one type of microplastic, excessively high exposure doses, and relatively short exposure durations. Future studies should design dose-response curves based on environmental monitoring data and human exposure levels, incorporating long-term, low-dose exposure models to better simulate real risks. Furthermore, research should fully consider factors such as the particle size, polymer type, weathered state, and surface adsorbed substances of microplastics, avoiding the simplistic use of laboratory-made polystyrene or polyethylene microspheres.

## Conclusion

As global plastic production continues to rise, MPs pollution in the environment is increasing exponentially. MPs are ubiquitous, contaminating the air, water, and food chain, leading to a higher likelihood of direct or indirect human exposure to MPs each year. Growing evidence from various models suggests that MPs are harmful to skin tissues, though the underlying mechanisms of damage remain unclear. To elucidate their impact, it is essential to deepen our understanding of how MPs disrupt both the gut and skin via the gut–skin axis.

This review first outlines the pathways of MPs exposure through the skin and intestinal absorption, emphasizing that nanoplastics can penetrate the stratum corneum or enter the body via endocytosis and paracellular routes in the intestinal epithelium, damaging both skin and intestinal epithelial barriers, inducing oxidative stress, and causing immune imbalance. Further analysis reveals that after accumulating in the gut, MPs disrupt the mucosal barrier and alter microbiota composition, leading to “dysbiosis.” This not only facilitates the translocation of exogenous endotoxins but also results in the reduced production of key metabolites. For instance, declines in butyrate‑producing bacteria (*Faecalibacterium and Akkermansia*) diminish short-chain fatty acid production, thereby weakening PPARγ-mediated keratinocyte differentiation and anti-inflammatory effects. Similarly, reductions in tryptophan-metabolizing bacteria (e.g., *Clostridium sporogenes and Bacteroides*) lower levels of indole-3‑aldehyde and other AHR ligands, impairing skin barrier repair and immune regulation. MPs also interfere with bile acid conversion, lowering secondary bile acids such as lithocholic acid and deoxycholic acid, which normally suppress NF‑κB and NLRP3 signaling via FXR/TGR5 and maintain Th17/Treg balance. Loss of these metabolites, coupled with excessive activation of innate immune pathways (e.g., TLR2–MyD88–NF‑κB), fosters skin inflammation, metabolic disturbances, and oxidative stress, ultimately disrupting skin immune homeostasis.

It is noteworthy that MPs may also directly damage the skin through local exposure. For instance, in an ADmodel, 70 nm particles were found to penetrate damaged skin and accumulate in the dermis. Thus, the toxicity of MPs includes both systemic effects mediated through gut microbiota and their metabolites, as well as local effects from direct skin penetration. This insight provides a theoretical basis for the potential applications of microbiota-based interventions and barrier protectants in preventing and treating microplastic-associated skin diseases and suggests that future research should focus on both the gut and skin as key targets.

## Supplementary Information

Below is the link to the electronic supplementary material.

Supplementary Material 1

## Data Availability

The analyzed data in this study are available from the corresponding author upon reasonable request.

## Abbreviations

AIM2: Absent in Melanoma 2
ACP: Acid phosphatase
AD: Atopic dermatitis
AKP: Alkaline phosphatase
ALT: Alanine Aminotransferase Increase
AST: Aspartate Aminotransferase Increase
AQP mRNA: Aquaporin mRNA
ATF4: Activating Transcription Factor 4
Arf1: ADP-Ribosylation Factor 1
ALA: Alpha-Linolenic Acid
ARA: Arachidonic Acid
AMP: Adenosine Monophosphate
ACP: Acid Phosphatase
AKP: Alkaline Phosphatase
AAs: Amino Acids
Acetyl-CoA: Acetyl Coenzyme A
AhRs: Aryl hydrocarbon receptors
AMP: Antimicrobial peptide
AhR: Aromatic hydrocarbon receptor
BAs: Bile Acids
B/F: Bacteroidetes/Firmicutes
COL1A1: Collagen Type I Alpha 1 Chain
C3: Complement Component 3
C4: Complement Component 4
CCR9B: C-C Motif Chemokine Receptor 9b (zebrafish homolog)
CAT: Catalase
COXIV: Cytochrome C Oxidase Subunit IV
CDH2: Cadherin-2
CXCL11: C-X-C Motif Chemokine Ligand 11
CLEC10A: C-Type Lectin Receptor 10 A
CHIT: Chitinase
CLEC17A: C-Type Lectin Domain Family 17
COL: Collagen
COL1A2: Collagen Type I Alpha 2 Chain
CD80: Cluster of Differentiation 80
Caspase-3: Cysteine-aspartic acid protease 3
CLEC10A: C-Type Lectin Domain Family 10 Member A
CMP: Cytidine Monophosphate
CDH2: Cadherin-2 (N-Cadherin)
CDCA: Chenodeoxycholic acid
DAO: Diamine oxidase
D-Lac: D-lactic acid
DHA: Docosahexaenoic Acid
ERK1: Extracellular Signal-Regulated Kinase 1
ECM: Extracellular matrix
FTH: Ferritin Heavy
FKBP1: FK506 Binding Protein 1
FXR: Farnesoid X receptor
GMP: Guanosine Monophosphate
GTP: Guanosine Triphosphate
GALT: Gut-associated lymphoid tissue
GPCRs: G-protein-coupled receptors
GSDMD: Gasdermin D
GC: Granulocyte
GST: Glutathione S-Transferase
GPCR: G-Protein-Coupled Receptor
HO-1: Heme Oxygenase-1
HDL: High-Density Lipoprotein
HYOU1: Hypoxia Up-Regulated 1
Hc: Hemolysin
HDAC: Histone Deacetylase
IgA: Immunoglobulin A
IFN-γ: Interferon-gamma
IL-6: Interleukin-6
IKKβ: IκB Kinase Beta
IBD: Inflammatory bowel disease
I3A: Indole-3-aldehyde
IPyr: Indole pyruvate
IPA: Indole-3-propionic acid
I3A: Indole-3-aldehyde
Klf4: Krüppel-Like Factor 4
Keap1: Kelch-Like ECH-Associated Protein 1
LAMA: Laminin Subunit Alpha
LPS: Lipopolysaccharide
LYZ: Lysozyme
LCA: Lithocholic acid
MALT: Mucosa-associated lymphoid tissue
MAIT: Mucosal-associated invariant T
MAC: Membrane attack complex
Mep1β: Metalloproteinase 1 Beta
MyD88: Myeloid Differentiation Primary Response 88
MPO: Myeloperoxidase
MCFD2: Multiple Coagulation Factor Deficiency Protein 2
MDA: Malondialdehyde
Muc2: Mucin 2
MMP9: Matrix Metalloproteinase 9
NRF1: Nuclear Respiratory Factor 1
Nrf2: Nuclear Factor Erythroid 2
NLRP3: NOD-, LRR- and pyrin domain-containing protein 3
Nrf2: Nuclear factor erythroid 2-related factor 2
NF-κBP65: Nuclear Factor kappa-light-chain-enhancer of activated B cells (p65 subunit)
OCLN: Occludin
PPO2: Prophenoloxidase
PIGR: Polymeric Immunoglobulin Receptor
PVEC: Pulmonary Vascular Endothelial Cells
PVEC: Portal Vein Endothelial Cell
PPAR: Peroxisome proliferator-activated receptor
PDCD6IP: Programmed Cell Death 6 Interacting Protein
PRRs: Pattern recognition receptors
ROS: Reactive Oxygen Species
RTN1: Reticulon 1
Retnlb: Resistin-Like Beta
SCFAs: Short-chain fatty acids
SOD: Superoxide dismutase
SGC: Semi-granulocyte
Serum TBA: Serum Total Bile Acids Increase
SPP1: Secreted Phosphoprotein 1
S. aureus: Staphylococcus aureus
SDA: Stearidonic Acid
SLE: Systemic lupus erythematosus
TLR: Toll-like receptor
THC: Total hemocyte count
TNF-α: Tumor Necrosis Factor-alpha
TLR4: Toll-like Receptor 4
TNFSF13: Tumor Necrosis Factor Superfamily Member 13
T-GSH: Total Glutathione
T-AOC: Total Antioxidant Capacity
Trx1: Thioredoxin 1
TXNDC5: Thioredoxin Domain Containing 5
TMA: Trimethylamine
TMAO: Trimethylamine N-oxide
TEWL: Transepidermal water loss
VEGF-A: Vascular Endothelial Growth Factor A
vWF: von Willebrand Factor
XBP-1 mRNA: X-box Binding Protein 1 mRNA
ZO-1: Zonula occludens-1
