# Supplementary material for: Emerging mechanisms of microplastic-induced skin diseases: a perspective from the gut–skin axis
Source: J Transl Med. 2026 Feb 16;24:257. doi: 10.1186/s12967-025-07300-w (PMC12911421; doi:10.1186/s12967-025-07300-w)
Supplement: Supplementary file 1 — Supplementary Material 1 [file 12967_2025_7300_MOESM1_ESM.docx]

Supplementary Table 1. Effects of MPs/NPs on Gut Microbiota and Their Immune Functions

| **Nanoplastic** | **Exposure Duration** | **Exposure Pathway** | **Species** | **Microbiome Changes** | **Mechanisms** | **Immune Effects** | **Metabolic Changes** | **Predictive Analysis** | **Reference** |
| --- | --- | --- | --- | --- | --- | --- | --- | --- | --- |
| **Vertebrates** |  |  |  |  |  |  |  |  |  |
| Polystyrene nano/microplastic particles  (50 μm, 100 nm) | 2 weeks | Aquaculture water | Wild-type zebrafish | *Phylum level:*  *Actinobacteri↓, Bacteroidete↓, Cyanobacter↓, Firmicutes↓, Proteobacter↓, and Spirochaetes↓.*  *Genus level:*  *Aurantimicrobium↓,*  *Cetobacteriu↓; Flavobacterium↑,*  *Mesorhizobium↑,*  *Mycobacterium↑and Bradyrihizobium↑.* | Intestinal cell damage, induced oxidative stress and inflammatory response, characterized by pre-immune responses and cytokine gene expression. | IL-10↓,IL-8↓,NF-κB↓，SOD↑, CAT↑, GSH-Px↑. | - | Present | [1] |
| Polystyrene MPs  (32–40 μm) | 28 days | Concentrations: 0, 100 μg/L (MP-100), 1000 μg/L (MP-1000) | Juvenile guppy (P. reticulata) | Phylum level:  Deformibacteria↑, Actinobacteri↓.  Family level:  Microbacteriaceae↓,  Rhizobiaceae↑  Genus level:  Gemmobacter↑, Rhodobacter.↓ | MPs reduced digestive enzyme activity and increased intestinal immune cytokine levels, inhibiting metabolic and repair pathways. | TNF-α↑, IFN-γ↑, TLR4↑, IL-6↑. | Decreased trypsin, chymotrypsin, amylase, and lipase activities. | MP-1000 may reduce cell cycle regulation, nucleotide transport and metabolism, translation, ribosome structure, cell movement, 3and d4efense mechanisms. Higher MPs concentrations may increase chromatin structure, cell movement, and extracellular structure pathways. | [2] |
| PE-MPs (1–1000 μg/mL) | 168 hours | Recirculating water | Zebrafish | Phylum level:  Firmicutes ↓, Bacteroidetes↓, Proteobacteria↑ Cyanobacteria↑.  Genus level:  Acinetobacter↑, Plesiomonas↑, Escherichia-Shigella↑,  Cetobacterium↓. | - | Exposure to T100 increased C4, whereas T1000 decreased C3 and C4; expression of TNFSF13, IL-4, PIGR, CD80, IL-10, CCR9B, CD28, CXCL12A, and IGHV4-5 significantly increased. | - | - | [3] |
| Polystyrene microplastics (PS-MPSs) | 8  weeks | Feed | Leuciscus waleckii | Phylum level:  Deformibacteria↑, Actinobacteria↑,  Firmicutes↑.  Genus level:  Rhodobacteraceae↓, Bacillus↓, Aurantimicrobium↑, Pseudorhodobacter↑. | PS-MPSs disrupted the physical barrier of the gut, activated NF-κB signaling, and suppressed Nrf2 signaling, leading to intestinal inflammation and oxidative stress. | MyD88↑, NF-κB P65↑, IKKb↑, Caspase 3↑, Caspase 8↑; Claudin-3↓, Claudin-f↓, Occludin-a↓, ZO-1↓; | Decreased digestive enzyme activity (lipase, trypsin, amylase). | - | [4] |
| MPS (8–12 µm) PE-MP (0, 1, 5, 10 mg/L) | 21 days | Aquaculture water | Mudfish  (P.dabryanus) | Phylum level:  *Deformibacteriass↑*, *Firmicutes↓*. | PE-MPs accumulated in tissues, causing histopathological changes and oxidative stress, disrupting the gut physical barrier and microbial balance. | DAO↑, D-Lac levels↑, GSH-Px; SOD and CAT activity were higher at 5 mg/L, decreased at 10 mg/L. | Increased ROS, lipid peroxidation, and intestinal histopathological damage. | - | [5] |
| Polystyrene nano/microplastics (PS-MNPs) | 21 days | Aquaculture water | Marine medaka |  |  | PPARs↓TNF-α↓IL-8↓COX1↓. | Decreased lipid synthesis genes (SREBF1, SREBF2, FASN, CPT) and metabolic changes. | - | [6] |
| Polystyrene microplastics (PS-MPs, 5 µm) | 5 weeks | Feed |  | Japanese quail | Phylum level:  *Firmicutes*↓,  *Deformibacteria*↓,  *Bacteroidetes*↑,  Genus level:  *Bacteroides*↑, *Streptococcus*↓*, Pediococcus*↓. | ROS↑, IL-1β↑, TNF-α↑, IL-6↑, IFN-γ↑, Claudin-1↓, Claudin-2↓, ZO-1↓. | - | - | [7] |
| PS-NPs (80 nm) | 5 days | Aquaculture water | Grass carp | Phylum level: Deformibacteria↑,  Firmicutes↑, Cetobacterium↓, Neisseria↓. | PS-NPs toxicity on gut health, alleviated by probiotics, improved gut morphology and balance. | CAT↑, SOD↑, GST↑,  MPO↑, MDA ↑, ROS ↑  , IL-6↑, IL-10↑, TNF-α↑, IFN-γ↑. | - | - | [8] |
| Polyethylene microplastic (PE-MP) with LCT | 21 days | Aquaculture water | Zebrafish | Phylum level: Deformibacteria↑, Firmicutes↑, Firmicutes↓. | PE-MPs enhanced LCT toxicity, induced oxidative stress, and altered immune responses. | CAT↑, MDA↑, SOD↓ , CAT↓, NF-κB↑, IL-1β↑,  TNF-α↑, tlr2↑, tlr4↑, myd88↑, nf-κb↑. |  |  | [9] |
| PS MP + PVC MP | 60 days | Oral gavage | Healthy male C57BL/6J mice | Bacteroidetes↓, Proteobacteria↓,  Firmicutes↑, Actinobacteria↑,  Patescibacteria↑, Lachnospiraceae_UCG-006↓,  Clostridium↓, Desulfovibrio↓,  Ruminococcaceae_unclassified↓, Erysipelatoclostridium↑. | ALT↑, AST↑, TNF-α↑, IL-1β↑, IL-6↑, T-AOC↓, U-GSH↓, SOD↓, MDA↑. | Disruption of intestinal mucosal barrier (↓ Muc2, Muc3, Klf4, Retnlb, Mep1β, Claudin-4/5, Zo-1); liver inflammation and lipid metabolism disorders. | ALT↑, AST↑, TNF-α↑, IL-1β↑, IL-6↑,   1. AOC↓, GSH↓, SOD↓, MDA↑,   Muc2↓, Muc3↓, Klf4↓, Retnlb↓, Mep1β↓, Claudin-4/5↓, Zo-1↓. | **Serum metabolomics:** Enriched (positive ion mode): thiamine metabolism, phenylacetic acid metabolism, phosphatidylethanolamine biosynthesis, alanine and butyrate metabolism. Enriched (negative ion mode): butyrate, thiamine, phenylacetic acid, D-arginine/D-ornithine metabolism, ketone bodies, bile acid biosynthesis. | [10] |
| 1.22 μm PS microplastics | 5 weeks | Feeding | Piglets | **Genus level** Candida↑,  utaneotrichosporon↑, Diutina↓. | Diarrhea incidence↑, intestinal barrier function↓; impaired intestinal angiogenesis via ROS/METTL3 pathway. | AQP mRNA↓, ZO-1↓,  Occludin↓, mucins ↓,  Keap1↓, HO-1↓, COXIV, β-F1-ATPase, NRF1 ↓, TGF-β1↓, VEGF-A↓,  MMP9↓, CDH2↓,  CXCL11↓, CASP1↓, PVEC↓, Nrf2↑, ROS↑, IL-1β↑, ATF4↑, ATF6↑, XBP-1 mRNA↑. | Digestive enzymes (lipase, lactase) ↓. | Melanogenesis pathway↑, Relaxin signaling pathway↑ | [11] |
| 0.5% or 5% PS-MPs (1-20 µm) | 21 days | Aquaculture water | Sparus aurata (Gilthead seabream) | C.propionicum↑, C. botulinum↑, L.antarcticum↑, E. rosea↑, M. luteus↑, P.bifermentans↓, O.hongkongensis↓. | Disrupted gut microbiota structure; Reduced core microbiota; Decreased major bacterial populations, Low abundance species. | TLR2/TLR5↑, COX-2↑. | Serum TBA↑, liver cholesterol↑, HDL ↑. |  | [12] |
| PE MPs (0.002 μg/g/d and 0.2 μg/g/d) | 30 days | Oral gavage | 36 Female 8-week-old ICR mice | Firmicutes ↓, Bacteroidetes↑. | Stimulated immune response;  Reducing mucin production. | ERK1↓, NF-κB↓, IL-1β↓, IL-8↑, IL-10↑. | ↑ Serum total protein, albumin, globulin. | Enhanced metabolic function, focusing on amino acid metabolism in gut microbiota. | [13] |
| Nano-PS (100 nm) | 14 days | Aerated seawater | Larimichthys crocea | **Phylum level**:  Bacteroidetes↑, Firmicutes ↑. **Genus level**:  Alloprevotella↑, Parabacteroide↑, Bifidobacterium↑, Alistipes↑, Bacteroides↑, Aliivibrio↑, Lactobacillus↑, Weissella↑ , Ruegeria↓, Vibrio↓, Microscilla↓. | Reduced immune  and digestive enzyme activities; impaired growth performance. | Lipase↓, Trypsin↓, Lysozyme activity↓. | - | Secondary metabolite biosynthesis pathways ↑; Circulatory system functions↓. | [14] |
| **invertebrate** |  |  |  |  |  |  |  |  |  |
| Low-density polyethylene (LDPE) (14 µm) | 28 days | Soil | Earthworm (Eisenia fetida) | Phylum level: *Deformibacteria↓Crenarchaeota↓*, *Bacteroidetes↓*, *Actinobacteria↑ Firmicutes↓*, Genus level:  *Adhaeribacter↓*, *Solirubrobacter↓*, *Sphingomonas↓*. | Increased toxicity in earthworms, resulting in weight loss and severe oxidative damage. Decreased alpha diversity and disrupted microbial community structure at the phylum and genus levels in both soil and earthworm intestines. Increased abundance of antibiotic-resistant genera. | SOD↑, CAT↑, GSH↑ during days 14 and 28, followed by a trend of inhibition. MDA↑. | - | - | [15] |
| Five common non-plasticized MPs: PE (6–18 μm), PTFE (1–8 μm), PP (1.77–18 μm), PS (100–200 μm), PVC (1–13 μm) | 14 days | Aquaculture water | Litopenaeus vannamei | Phylum level:  *Bacteroidetes↑*, *Proteobacteria↑*, *Firmicutes↓*.  Genus level:  *Alteromonadales↑*, *Flavirhabdus↑*, *Formosa↑*, *Halocynthiibacter↑*, *Pseudoalteromonas↑*, *Rhodobacteraceae↑*, *Ruegeria↑*, *Tamlana↑*, *Tenacibaculum ↑*. | Altered gut microbiota composition (Bacteroidetes/Proteobacteria↑, Firmicutes↓); reduced lactic acid and short-chain fatty acid producers; disrupted lipid metabolism and immune-related protein  expression. | - PE group, HYOU1↑ RhoA↑, CLEC10A↓ -PS group, HYOU1↑, CLEC17A↑, RhoA↑, Arf1↑, vWF↑,  Trx1↓,CLEC10A↓, CHIT↓.  -PVC group, SERPINB↑ α2M↑, GST↑, TXNDC5↑, CLEC17A↑, PDCD6IP↑, MCFD2↑, CaM↑, RTN1↑, LAMA↑, FTH↓, CLEC10A ↓.  - PTFE group：PDCD6IP↑, FKBP1↑.  -PP group：CHIT↓, COL↓. | 1.Hemolymph metabolomics (amino acids): - PE/PS/PVC/PTFE: Valine, leucine, isoleucine, phenylalanine, tyrosine, tryptophan biosynthesis/metabolism altered - PP: Taurine/hypotaurine, glycine, serine, threonine metabolism altered - PS/PTFE: D-glutamine/D-glutamate metabolism disrupted. 2. Lipid dysregulation: ALA pathway altered; ARA, DHA, SDA, ALA levels ↓. 3. Energy metabolism: L-malate levels ↓. 4. Nucleotide metabolism: Thymidine ↑; AMP, GMP, CMP, GTP ↓. | KEGG pathways: - PE: T cell receptor, Wnt, colorectal cancer pathways - PS: T cell receptor, Helicobacter pylori infection, phospholipase D signaling - PVC: Unsaturated fatty acid biosynthesis, phosphatidylinositol signaling, RNA degradation - PTFE: Unsaturated fatty acid biosynthesis, tryptophan/nicotinate metabolism - PP: Ganglio-/isoglobo-series glycosphingolipid biosynthesis, glycosaminoglycan degradation. | [16] |
| Polystyrene microspheres (25 mg/mL; 5 μm) | 21 days | Aquaculture water | Eriocheir sinensis | Phylum level:  *Cyanobacteria↑*, *Chloroflexi↑*, *Fusobacteria↑*, *Proteobacteria↑*, *Nitrospirae↓*, *Bacteroidetes↓*, *Firmicutes↓*. | Enhanced immune enzyme activity and immune-related gene expression. | Hc↓, LSZ↓, MyD88↑, ACP↑, AKP↑. | - | - | [17] |
| PE | 21 days | Aquaculture water | Procambarus clarkii (crayfish) | Bacteroidaceae↓, Lachnospiraceae↓, Erysipelotrichaceae↓Aeromonadaceae↓, Dysgonomonadaceae↓, Shewanellaceae↑, Enterobacteriaceae↑, Mycoplasmataceae↑, Vibrionaceae↑. | Upregulation of immune-related genes, including transcription factors and antimicrobial peptides. | GSH-PX↓, NO↓, MDA↓,  SOD↑, H2O2↑,  THC↓, GC↓, SGC↓. | - | - | [18] |
| 2 μm monodisperse green fluorescent PS microbeads | (21 days) | Nutrient solution | P. versicolora (leaf beetle larvae) | **Phylum:** *Proteobacteria↑*,  *Actinobacteria↑*, *Firmicutes↑*,  **Genus:** Pseudomonas↑, Rothia↑, Pantoea↑, Enterobacter↑. | MNP intake activated detoxification, apoptosis, and energy metabolism; suppressed digestion, gut homeostasis, and immune function. | Genes in: carbohydrate, lipid, amino acid metabolism; xenobiotic degradation; apoptosis  ↓ Genes in: adrenergic signaling, cardiac contraction, cholesterol metabolism, Hippo/FoxO pathways, Toll/Imd pathways, mucin-type O-glycan biosynthesis. | - | - | [19] |
| 75 nm PS-NPs (2.5% w/v)  Exposure concentrations: 2.5, 5, 10, 20 mg/L | 7 days | PS-NP solution | Eriocheir sinensis (Chinese mitten crab) | Shewanella marisflavi↑, Haemophilus parainfluenzae↑, Ralstonia insidiosa↑, Solobacterium moorei↑, Actinomyces graevenitzii↑, Rothia mucilaginosa↑, Porphyromonas gingivalis↑, Prevotella intermedia↑, Porphyromonas endodontalis↑, Campylobacter spp↑. | Altered immune function and tissue damage. | Myd88↑, Caspase↑, LITAF↑, Hc↑. | SOD↓, POD↓, GPX  activity↓; MDA↑, GSH↑. |  | [20] |
| 200 nm PS microspheres (concentrations: 0, 0.5, 5 mg/L) | 14 days | Hydroponic system | Cherax quadricarinatus (Red crayfish) | **Phylum level:** Cyanobacteria↑,  **Genus level:** Bacteroidetes↓, Lactobacillus↓, Lachnospiraceae NK4A136↓,  Dubosiella↓,  Ruminiclostridium↓. | Induced oxidative stress and lipid metabolism disruption; Low concentration: immune response; High concentration: immune gene expression. | SOD↑, CAT↑ activity at 25 μg/L; decline at 250 and 2500 μg/L  LYZ, PPO2 ascend at 25 μg/L; decline at 250 and 2500 μg/L. | - | Metabolism, human diseases, organism systems, genetic information, cellular processes, environmental information processing. | [21] |
| PS MPs (100 nm, 1 μm, 10 μm, Fluorescent PS-10 μm) | 15 days | Feeding | Honeybees (Apis mellifera) | Lactobacillus↓. | Disrupted gut microbiota, reducing nutrient absorption from pollen, causing gut underdevelopment and inflammation, leading to decreased digestive capacity. | MAPK↑. | - | Inflammatory gene expression upregulated, including protein phosphatases, MAPK pathway, ion channels, Rap1 signaling, and thyroid hormone signaling.  **Downregulated genes** related to detoxification, appetite, and glucose/lipid metabolism, including cytochrome P450, AMPK, and PPAR pathways. | [22] |

Supplementary Table 2. Pathogenic Mechanisms by Which Alterations in Gut Microbiota and Their Metabolites Influence Skin Diseases

| **Disease** | **Gut Microbiota Alterations** | **Gut Metabolites** | **Pathogenic Mechanisms** | **Reference** |
| --- | --- | --- | --- | --- |
| **Atopic Dermatitis** | *Firmicutes↑*  *Actinobacteria↑*  *Bacteroidetes↑*  *Proteobacteria↑ Bacteroidales↑ Romboutsia↑*  *Sutterella↑*  *Akkermansia↓*  *Lactobacillus↓*  *Blautia↓*  *Bifidobacterium↓ Clostridium↑*   1. *coli↑*   *S. aureus↑* | SCFAs ↓  (Butyrate, propionate), Treg↓;  Tryptophan↑ | Butyrate/propionate inhibit mast cell degranulation; butyrate promotes keratinocyte differentiation and barrier repair; tryptophan metabolites activate AHR to enhance filaggrin expression; FICZ reverses IL-4-induced filaggrin suppression | [23,24] |
| **Psoriasis** | *Bacteroidota↓*  *Firmicutes/Bacteroidetes↑ ratio,*  *Proteobacteria↑*  *Prevotellaceae↓ Ruminococcaceae↑ Lachnospiraceae↑ Butyricicoccus↓ Lachnoclostridium↓*  *Alistipes↓*  *Agathobacter↓*  *Roseburia hominis↓ Escherichia spp↑* | Succinate↑, pro-inflammatory ligands↑,  TMA↑;  SCFAs↓,  Tryptophan↑, Secondary BAs↑ | SCFAs modulate GPCR/HDAC, restore Th17/Treg balance, and maintain gut barrier integrity | [25] [25] [26] [26] |
| **Acne** | *Firmicutes↓*  *Bacteroidetes↓ Bifidobacterium↓ Lactobacillus↓ Ruminococcaceae↓ Actinobacteria↓*  *Proteobacteria↑*  *Clostridium↓ Lachnospiraceae↓ Clostridium sensu stricto↑* | 1. phenylglycine↑, D-2-hydroxyvaleric acid↑,   various AAs↑, putrescine↑; 3-hydroxyisovalerate↓ | Probiotics enhance barrier, suppress T/B cell activation; Ruminococcus protective; Allisonella, Bacteroides exacerbate acne | [27] [28] [27] |
| **Rosacea** | *Veillonellaceae↓ Methanobrevibacter↓ Aminopeptococcus↑ Megasphaera↑* | — | — |  |
| **Alopecia (AA)** | *Firmicutes↓*  *Proteobacteria↓ Actinobacteria↓*  *Clostridium↓*  *Bifidobacterium↓*  *Streptococcus↓*  *Butyricimonas↓*  *Enterorhabdus↓*  *Muribaculaceae↑*  *Parabacteroides* | SCFAs↓ | Ruminococcaceae may impair immune regulation and barrier via SCFA reduction | [29][30]  [31] |
| **Melanoma** | *Actinobacteria↓*  *Firmicutes↓*  *Prevotella7↑ Parabacteroides↑ Cyanobacteria↑ Ruminococcaceae UCG013↓* | MC-LR↑  Butyrate esters↓  Propionate esters↓ | MC-LR promotes TGF-β1 in M2 macrophages and suppresses CST3 in cancer cells | [32] [33] [34][35] |
| **Skin Aging** | *Bifidobacterium↓ Bacteroides↓ Enterobacteriaceae↓*  *Clostridium↑*  *Bacteroides spp↑* | Acetyl-CoA↑,  Leucine and niacin degradation↑; Taxadiene biosynthesis↓,  tRNA processing↓, isoleucine synthesis↓ | Metabolites trigger SASP via ↑TNF-α, IFN-γ, IL-1, IL-6, MMPs, contributing to inflammaging | [36] [37] [38] [39]  [40] |
| **systemic lupus erythematosus (SLE)** | *Bacteroidota↑ Actinobacteria↑ Proteobacteria↑*  *Firmicutes↓*  *Prevotella↑*  *Parabacteroides↑*  *Klebsiella↑*  *Dialister↓*  *Pseudobutyrivibrio↓*  *Ruminococcaceae↓ Streptococcus↑*  *Veillonella↑* | Indole-3-propionic acid↑,  BAs↑; 2-hydroxyisobutyrate↑, glutamate↑;  Citrate↓,  glycerol↓,  linoleic acid↓ | Gut permeability via zonulin; microbiota correlates with Treg/Th17 balance and cytokine expression | [41] [42] [43] |

REFRENCE

1. Pei X, Heng X, Chu W. Polystyrene nano/microplastics induce microbiota dysbiosis, oxidative damage, and innate immune disruption in zebrafish. Microb Pathog. 2022;163:105387. https://doi.org/10.1016/j.micpath.2021.105387

2. Huang J-N, Wen B, Zhu J-G, Zhang Y-S, Gao J-Z, Chen Z-Z. Exposure to microplastics impairs digestive performance, stimulates immune response and induces microbiota dysbiosis in the gut of juvenile guppy (Poecilia reticulata). Sci Total Environ. 2020;733:138929. https://doi.org/10.1016/j.scitotenv.2020.138929

3. Yuan Y, Sepúlveda MS, Bi B, Huang Y, Kong L, Yan H, et al. Acute polyethylene microplastic (PE-MPs) exposure activates the intestinal mucosal immune network pathway in adult zebrafish (*Danio rerio*). Chemosphere. 2023;311:137048. https://doi.org/10.1016/j.chemosphere.2022.137048

4. Liu SY, Li DL, Zhu R, Meng ST, Wang YT, Li L, et al. Mechanisms underlying mitochondrial dysfunction and intestinal damage induced by ingestion of microplastics in *Leuciscus waleckii*: The role of the NF-κB/Nrf2 signaling pathway. Chemosphere. 2024;367:143676. https://doi.org/10.1016/j.chemosphere.2024.143676

5. Xia X, Ma X, Liang N, Qin L, Huo W, Li Y. Damage of polyethylene microplastics on the intestine multilayer barrier, blood cell immune function and the repair effect of *Leuconostoc mesenteroides* DH in the large-scale loach (*Paramisgurnus dabryanus*). Fish & Shellfish Immunology. 2024;147:109460. https://doi.org/10.1016/j.fsi.2024.109460

6. Liu L, Ma Y, Xu Y, Liu B, Wang C, Feng J, et al. Mechanisms of eco-corona effects on micro(nano)plastics in marine medaka: Insights into translocation, immunity, and energy metabolism. Journal of Hazardous Materials. 2024;480:136236. https://doi.org/10.1016/j.jhazmat.2024.136236

7. Jing L, Zhang Y, Zhang Q, Zhao H. Polystyrene microplastics disrupted physical barriers, microbiota composition and immune responses in the cecum of developmental Japanese quails. Journal of Environmental Sciences. 2024;144:225–35. https://doi.org/10.1016/j.jes.2023.08.020

8. Li Z, Chen F, Liu J, Zhi L, Junaid M, Chen G, et al. Polystyrene nanoplastics sequester the toxicity mitigating potential of probiotics by altering gut microbiota in grass carp (*Ctenopharyngodon idella*). Journal of Hazardous Materials. 2025;484:136778. https://doi.org/10.1016/j.jhazmat.2024.136778

9. Zhao Y, Chen H, Liang H, Zhao T, Ren B, Li Y, et al. Combined toxic effects of polyethylene microplastics and lambda-cyhalothrin on gut of zebrafish (*Danio rerio*). Ecotoxicology and Environmental Safety. 2024;276:116296. https://doi.org/10.1016/j.ecoenv.2024.116296

10. Zhuang J, Chen Q, Xu L, Chen X. Combined exposure to polyvinyl chloride and polystyrene microplastics induces liver injury and perturbs gut microbial and serum metabolic homeostasis in mice. Ecotoxicol Environ Saf. 2023;267:115637. https://doi.org/10.1016/j.ecoenv.2023.115637

11. Zou D, Yang Y, Ji F, Lv R, Wu H, Hou G, et al. Polystyrene Microplastics Causes Diarrhea and Impairs Intestinal Angiogenesis through the ROS/METTL3 Pathway. J Agric Food Chem. 2024;72:16638–50. https://doi.org/10.1021/acs.jafc.4c03238

12. Del Piano F, Mateu B, Coretti L, Borrelli L, Piccolo G, Addeo NF, et al. Polystyrene microplastic exposure modulates gut microbiota and gut-liver axis in gilthead seabream (Sparus aurata). Sci Total Environ. 2024;957:177857. https://doi.org/10.1016/j.scitotenv.2024.177857

13. Sun H, Chen N, Yang X, Xia Y, Wu D. Effects induced by polyethylene microplastics oral exposure on colon mucin release, inflammation, gut microflora composition and metabolism in mice. Ecotoxicol Environ Saf. 2021;220:112340. https://doi.org/10.1016/j.ecoenv.2021.112340

14. Gu H, Wang S, Wang X, Yu X, Hu M, Huang W, et al. Nanoplastics impair the intestinal health of the juvenile large yellow croaker *Larimichthys crocea*. Journal of Hazardous Materials. 2020;397:122773. https://doi.org/10.1016/j.jhazmat.2020.122773

15. Zhang Y, Qin K, Liu C. Low-density polyethylene enhances the disturbance of microbiome and antibiotic resistance genes transfer in soil-earthworm system induced by pyraclostrobin. J Hazard Mater. 2024;465:133459. https://doi.org/10.1016/j.jhazmat.2024.133459

16. Duan Y, Xiong D, Wang Y, Zhang Z, Li H, Dong H, et al. Toxicological effects of microplastics in *Litopenaeus vannamei* as indicated by an integrated microbiome, proteomic and metabolomic approach. Science of The Total Environment. 2021;761:143311. https://doi.org/10.1016/j.scitotenv.2020.143311

17. Liu Z, Yu P, Cai M, Wu D, Zhang M, Chen M, et al. Effects of microplastics on the innate immunity and intestinal microflora of juvenile Eriocheir sinensis. Sci Total Environ. 2019;685:836–46. https://doi.org/10.1016/j.scitotenv.2019.06.265

18. Zhang X, Jin Z, Shen M, Chang Z, Yu G, Wang L, et al. Accumulation of polyethylene microplastics induces oxidative stress, microbiome dysbiosis and immunoregulation in crayfish. Fish Shellfish Immunol. 2022;125:276–84. https://doi.org/10.1016/j.fsi.2022.05.005

19. Zhu P, Zhang Y, Deng M, Zhang Y, Luo J, Han R, et al. Microplastics and Nanoplastics Alter the Physicochemical Properties of Willow Trees and Lead to Mortality in Leaf Beetle Larvae. Plant Cell Environ. 2025;48:2895–909. https://doi.org/10.1111/pce.15317

20. Han M, Zhu C, Tang S, Liang J, Li D, Guo Y, et al. The effects of a polystyrene nanoplastic on the immune response and gut microbiota of Eriocheir sinensis and its post-recovery state. Aquat Toxicol. 2023;262:106644. https://doi.org/10.1016/j.aquatox.2023.106644

21. Cheng H, Dai Y, Ruan X, Duan X, Zhang C, Li L, et al. Effects of nanoplastic exposure on the immunity and metabolism of red crayfish (Cherax quadricarinatus) based on high-throughput sequencing. Ecotoxicol Environ Saf. 2022;245:114114. https://doi.org/10.1016/j.ecoenv.2022.114114

22. Wang K, Zhu L, Rao L, Zhao L, Wang Y, Wu X, et al. Nano- and micro-polystyrene plastics disturb gut microbiota and intestinal immune system in honeybee. Sci Total Environ. 2022;842:156819. https://doi.org/10.1016/j.scitotenv.2022.156819

23. Ye S, Yan F, Wang H, Mo X, Liu J, Zhang Y, et al. Diversity analysis of gut microbiota between healthy controls and those with atopic dermatitis in a Chinese population. J Dermatol. 2021;48:158–67. https://doi.org/10.1111/1346-8138.15530

24. Melli LCFL, Carmo-Rodrigues MS do, Araújo-Filho HB, Mello CS, Tahan S, Pignatari ACC, et al. Gut microbiota of children with atopic dermatitis: Controlled study in the metropolitan region of São Paulo, Brazil. Allergol Immunopathol (Madr). 2020;48:107–15. https://doi.org/10.1016/j.aller.2019.08.004

25. Dei-Cas I, Giliberto F, Luce L, Dopazo H, Penas-Steinhardt A. Metagenomic analysis of gut microbiota in non-treated plaque psoriasis patients stratified by disease severity: development of a new Psoriasis-Microbiome Index. Sci Rep. 2020;10:12754. https://doi.org/10.1038/s41598-020-69537-3

26. Wen C, Pan Y, Gao M, Wang J, Huang K, Tu P. Altered gut microbiome composition in nontreated plaque psoriasis patients. Microb Pathog. 2023;175:105970. https://doi.org/10.1016/j.micpath.2023.105970

27. Yan H-M, Zhao H-J, Guo D-Y, Zhu P-Q, Zhang C-L, Jiang W. Gut microbiota alterations in moderate to severe acne vulgaris patients. J Dermatol. 2018;45:1166–71. https://doi.org/10.1111/1346-8138.14586

28. Cao Q, Guo J, Chang S, Huang Z, Luo Q. Gut microbiota and acne: A Mendelian randomization study. Skin Res Technol. 2023;29:e13473. https://doi.org/10.1111/srt.13473

29. Moreno-Arrones OM, Serrano-Villar S, Perez-Brocal V, Saceda-Corralo D, Morales-Raya C, Rodrigues-Barata R, et al. Analysis of the gut microbiota in alopecia areata: identification of bacterial biomarkers. J Eur Acad Dermatol Venereol. 2020;34:400–5. https://doi.org/10.1111/jdv.15885

30. Lu J, Zhang P, Hu R, Qi S, Zhao Y, Miao Y, et al. Gut microbiota characterization in Chinese patients with alopecia areata. J Dermatol Sci. 2021;102:109–15. https://doi.org/10.1016/j.jdermsci.2021.04.003

31. Rangu S, Lee J-J, Hu W, Bittinger K, Castelo-Soccio L. Understanding the Gut Microbiota in Pediatric Patients with Alopecia Areata and their Siblings: A Pilot Study. JID Innov. 2021;1:100051. https://doi.org/10.1016/j.xjidi.2021.100051

32. Witt RG, Cass SH, Tran T, Damania A, Nelson EE, Sirmans E, et al. Gut Microbiome in Patients With Early-Stage and Late-Stage Melanoma. JAMA Dermatol. 2023;159:1076–84. https://doi.org/10.1001/jamadermatol.2023.2955

33. Vandoni G, D’Amico F, Fabbrini M, Mariani L, Sieri S, Casirati A, et al. Gut Microbiota, Metabolome, and Body Composition Signatures of Response to Therapy in Patients with Advanced Melanoma. Int J Mol Sci. 2023;24:11611. https://doi.org/10.3390/ijms241411611

34. Wu Z, Zhang S, Li L, Huang Z, Huang D, Hu Y. The gut microbiota modulates responses to anti-PD-1 and chemotherapy combination therapy and related adverse events in patients with advanced solid tumors. Front Oncol. 2022;12:887383. https://doi.org/10.3389/fonc.2022.887383

35. Limeta A, Ji B, Levin M, Gatto F, Nielsen J. Meta-analysis of the gut microbiota in predicting response to cancer immunotherapy in metastatic melanoma. JCI Insight. 2020;5:e140940, 140940. https://doi.org/10.1172/jci.insight.140940

36. Ratanapokasatit Y, Laisuan W, Rattananukrom T, Petchlorlian A, Thaipisuttikul I, Sompornrattanaphan M. How Microbiomes Affect Skin Aging: The Updated Evidence and Current Perspectives. Life (Basel). 2022;12:936. https://doi.org/10.3390/life12070936

37. Drago L, Toscano M, Rodighiero V, De Vecchi E, Mogna G. Cultivable and pyrosequenced fecal microflora in centenarians and young subjects. J Clin Gastroenterol. 2012;46 Suppl:S81-84. https://doi.org/10.1097/MCG.0b013e3182693982

38. Biagi E, Nylund L, Candela M, Ostan R, Bucci L, Pini E, et al. Through ageing, and beyond: gut microbiota and inflammatory status in seniors and centenarians. PLoS One. 2010;5:e10667. https://doi.org/10.1371/journal.pone.0010667

39. Min M, Egli C, Sivamani RK. The Gut and Skin Microbiome and Its Association with Aging Clocks. Int J Mol Sci. 2024;25:7471. https://doi.org/10.3390/ijms25137471

40. Chen M, Che Y, Liu M, Xiao X, Zhong L, Zhao S, et al. Genetic insights into the gut microbiota and risk of facial skin aging: A Mendelian randomization study. Skin Res Technol. 2024;30:e13636. https://doi.org/10.1111/srt.13636

41. Yao K, Xie Y, Wang J, Lin Y, Chen X, Zhou T. Gut microbiota: a newly identified environmental factor in systemic lupus erythematosus. Front Immunol. 2023;14:1202850. https://doi.org/10.3389/fimmu.2023.1202850

42. Wang X, Shu Q, Song L, Liu Q, Qu X, Li M. Gut Microbiota in Systemic Lupus Erythematosus and Correlation With Diet and Clinical Manifestations. Front Med (Lausanne). 2022;9:915179. https://doi.org/10.3389/fmed.2022.915179

43. Zhang S-X, Wang J, Chen J-W, Zhang M-X, Zhang Y-F, Hu F-Y, et al. The level of peripheral regulatory T cells is linked to changes in gut commensal microflora in patients with systemic lupus erythematosus. Ann Rheum Dis. 2021;80:e177. https://doi.org/10.1136/annrheumdis-2019-216504
